# Supplementary material for: The Effect of Processing Route on Properties of HfNbTaTiZr High Entropy Alloy
Source: Materials (Basel). 2019 Dec 3;12(23):4022. doi: 10.3390/ma12234022 (PMC6926650; doi:10.3390/ma12234022)
Supplement: Supplementary file 1 [file materials-12-04022-s001.pdf]

materials

MDPI

Article

The Effect of Processing Route on Properties of HfNbTaTiZr High Entropy Alloy

Jaroslav Málek [1,2,*], Jiří Zýka [1], František Lukáč [3,4], Monika Vilémová [3], Tomáš Vlasák [4], Jakub Čížek [4], Oksana Melikhova [4] Adéla Macháčková [5] and Hyoung Seop Kim [6]

1 UJP PRAHA a.s., Nad Kamínkou 1345, 156 10 Prague-Zbraslav, Czech Republic; zyka@ujp.cz (J.Z.)
2 CTU in Prague-Faculty of Mechanical Engineering, Karlovo Náměstí 13, 121 35 Praha 2, Czech Republic
3 Institute of Plasma Physics CAS, 182 00 Praha 8, Czech Republic; lukac@ipp.cas.cz (F.L.); vilemova@ipp.cas.cz (M.V.).
4 Faculty of Mathematics and Physics, Charles University, 180 00 Praha 8, Czech Republic; jakub.cizek@mff.cuni.cz (J.C.); tomas.vlasak@seznam.cz (T.V.); Oksana.Melikhova@mff.cuni.cz (O.M.).
5 Faculty of Materials Science and Technology, VŠB-Technical University of Ostrava, 17. Listopadu 15, 708 33 Ostrava 8, Cech Republic; adela.machackova@vsb.cz (A.M.)
6 Department of Materials Science and Engineering, POSTECH, Pohang, 790-784, South Korea; hyoungseopkim@gmail.com (H.K.)
* Correspondence: jardamalek@seznam.cz (J.M.)

Received: 5 November 2019; Accepted: 27 November 2019; Published: date

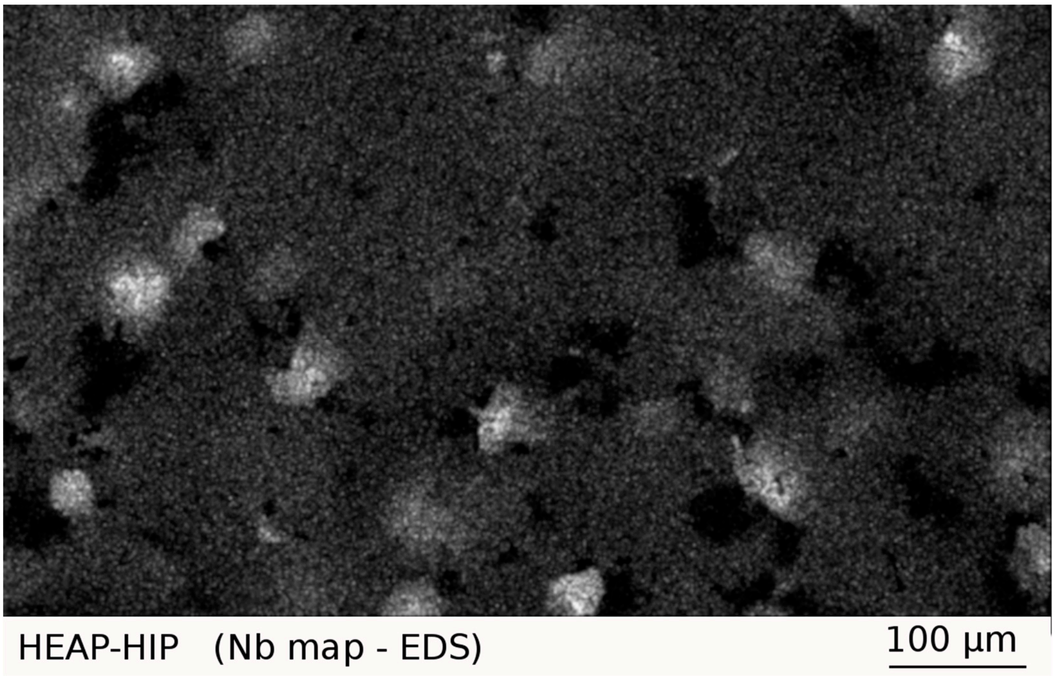

(a)

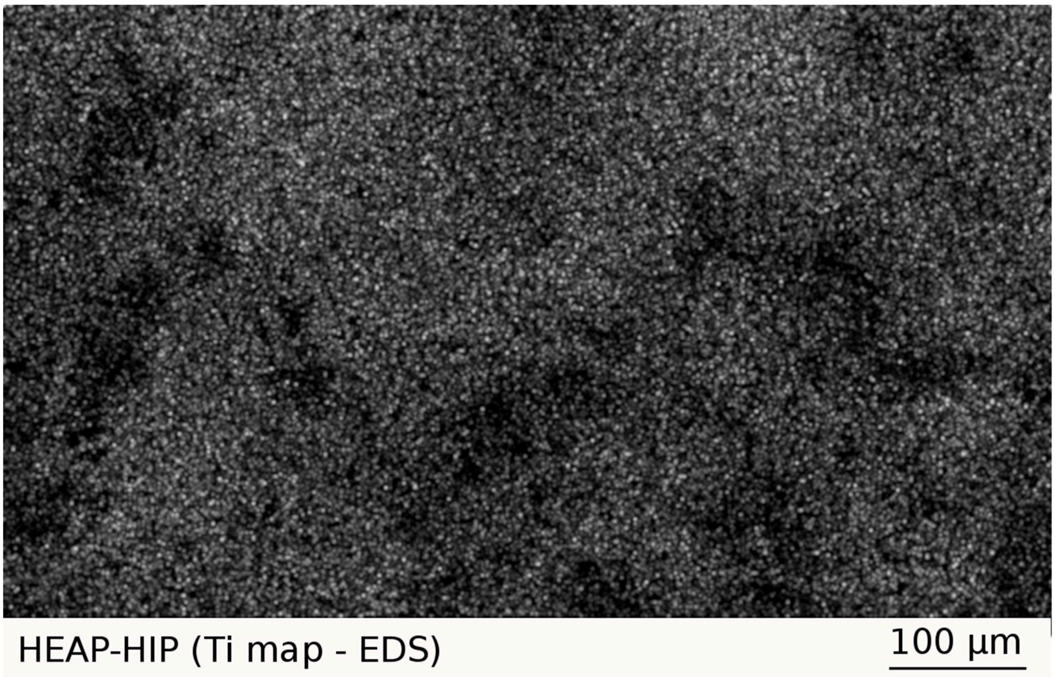

(b)

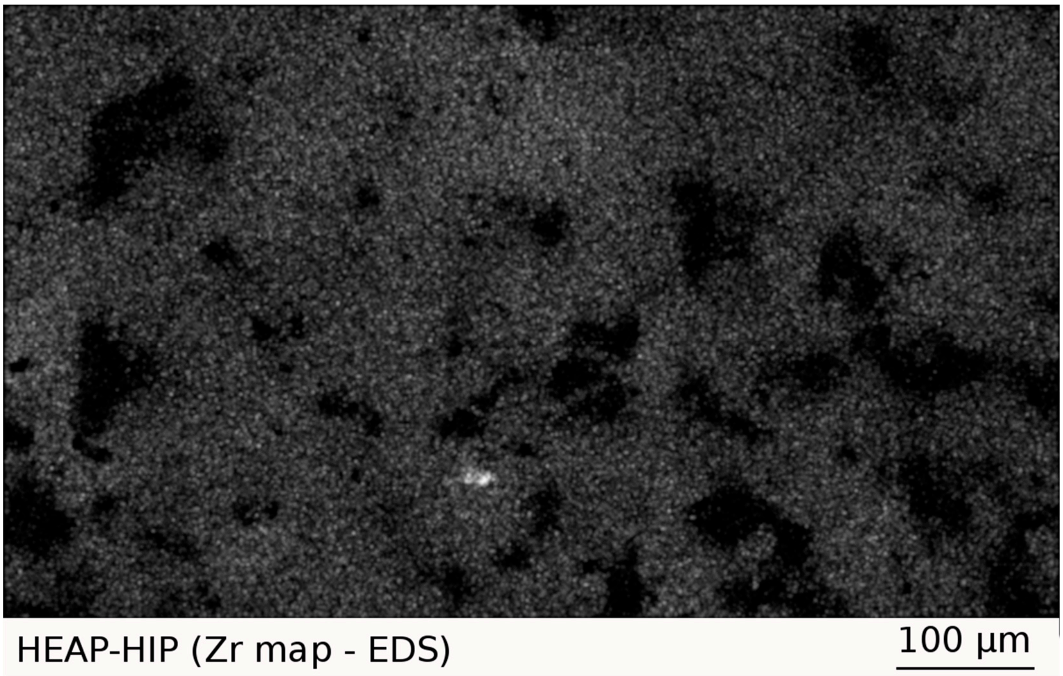

(c)

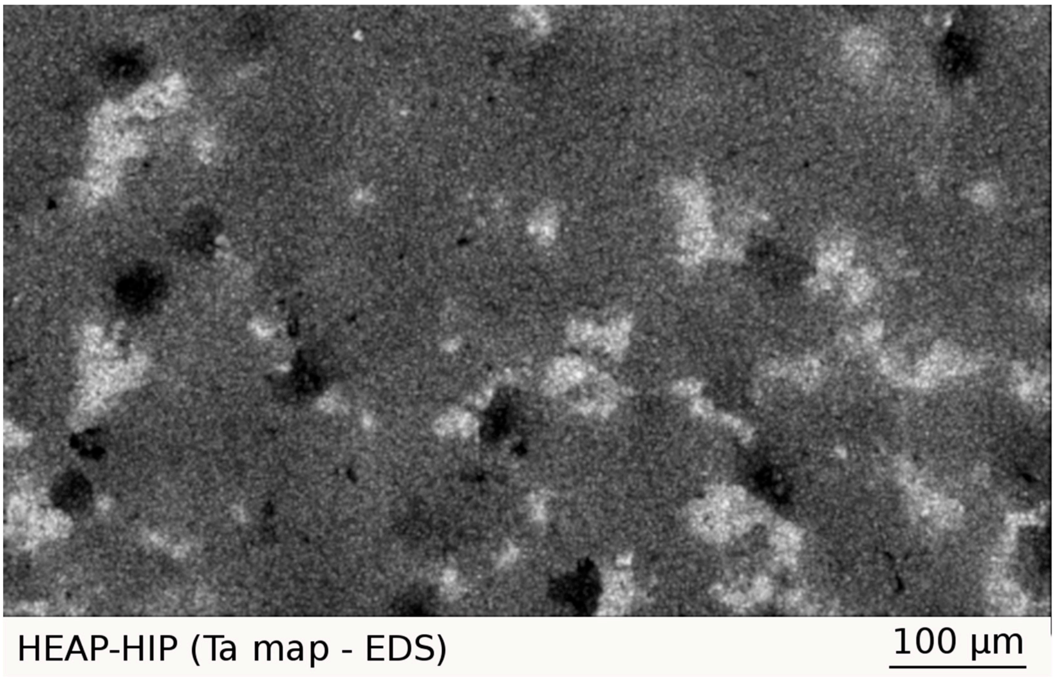

(d)

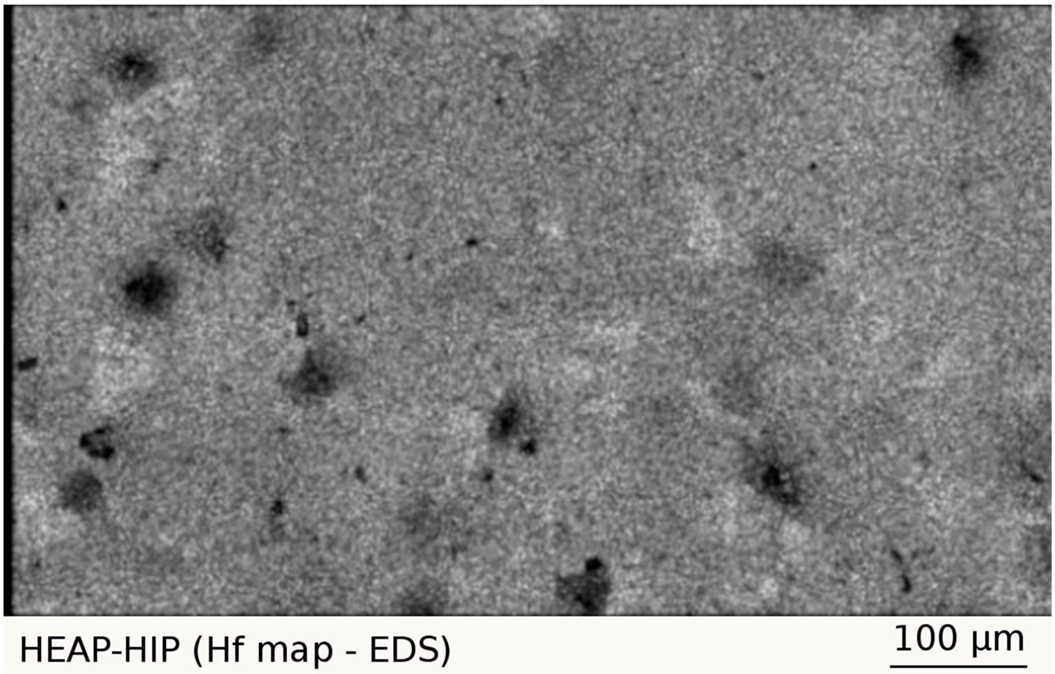

(e)

Figure S1. (**a**) Nb; (**b**) Ti; (**c**) Zr; (**d**) Ta; (**e**): Hf.
